# Supplementary material for: Chirality locking charge density waves in a chiral crystal
Source: Nat Commun. 2022 May 25;13:2914. doi: 10.1038/s41467-022-30612-0 (PMC9133074; doi:10.1038/s41467-022-30612-0)
Supplement: Supplementary file 1 — Supplementary Information [file 41467_2022_30612_MOESM1_ESM.pdf]

Supplementary Information

Chirality locking charge density waves in a chiral crystal

Geng Li[1,2,3,4#], Haitao Yang[1,2#], Peijie Jiang[1,2#], Cong Wang[5#], Qiuzhen Cheng[1,2], Shangjie Tian[5], Guangyuan Han[1,2], Chengmin Shen[1,2], Xiao Lin[1,2], Hechang Lei[5*], Wei Ji[5*], Ziqiang Wang[6*], Hong-Jun Gao[1,2,3,4*]

[1] Institute of Physics, Chinese Academy of Sciences, Beijing 100190, China

[2] School of Physical Sciences, University of Chinese Academy of Sciences, Beijing 100190, China

[3] CAS Center for Excellent in Topological Quantum Computation, University of Chinese Academy of Sciences, Beijing 100190, China

[4] Songshan Lake Materials Laboratory, Dongguan, Guangdong 523808, PR China

[5] Beijing Key Laboratory of Optoelectronic Functional Materials & Micro-Nano Devices, Department of Physics, Renmin University of China, Beijing 100872, PR China

[6] Department of Physics, Boston College, Chestnut Hill, MA, USA

[#]These authors contributed equally to this work.

[*]Correspondence to: hjgao@iphy.ac.cn; wangzi@bc.edu; wji@ruc.edu.cn; hlei@ruc.edu.cn

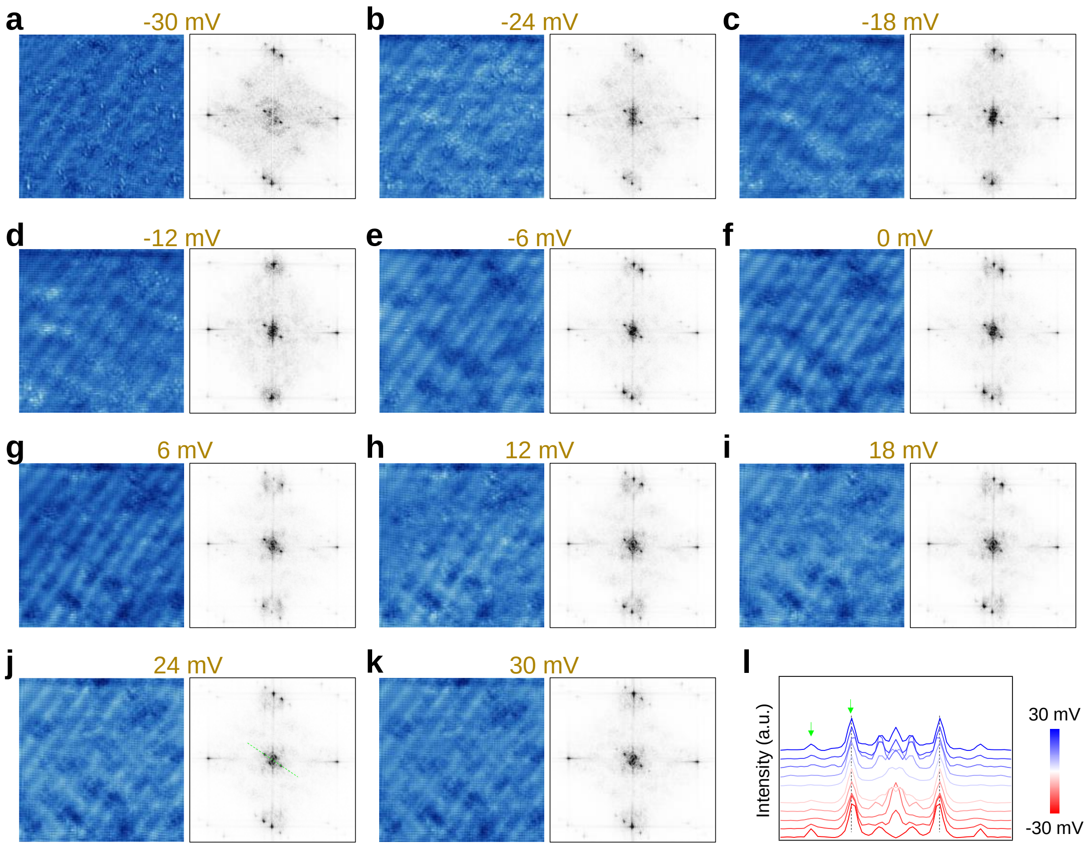

Supplementary Fig. 1: dI/dV maps under different bias voltages at the region presented in Fig. 2a of the main text. a-k, Left panels: dI/dV maps taken from -30 mV to 30 mV. Scanning area: 30 nm × 30 nm. Right panels: corresponding FT images of the left panels. **l,** Intensity curves along the Γ-Q_{CDW} direction (highlighted by green dashed line in **j**) taken from all the FT images. The curves are vertically offset for clarity. The green arrows highlight the position of the 1st and 2nd order of the CDW wave vector.

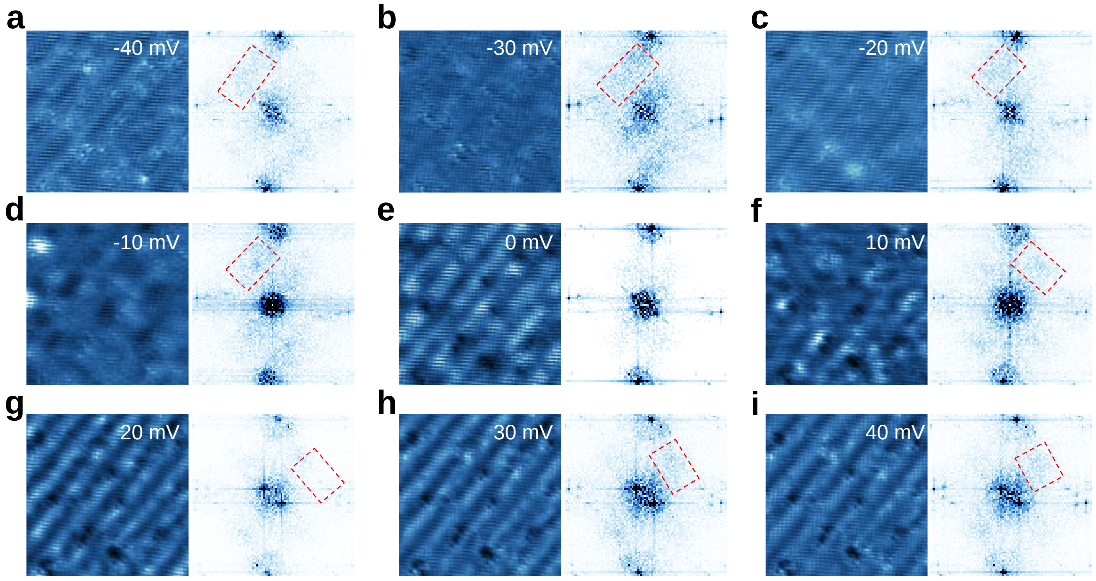

Supplementary Fig. 2: dI/dV maps under different bias voltages and corresponding FT images at another region. a-i, Left panels: dI/dV maps taken from -40 mV to 40 mV of a 20 nm × 20 nm area. Right panels: corresponding FT images of the dI/dV maps. Away from the Fermi energy, the CDW pattern can be resolved with no energy dispersion (**a-c, g-i**). Inside the CDW gap, the stripe pattern is suppressed by the in-gap states which have not been fully gapped out. The quasi-particle interference pattern is highly energy-dependent, as marked by the red dashed rectangles.

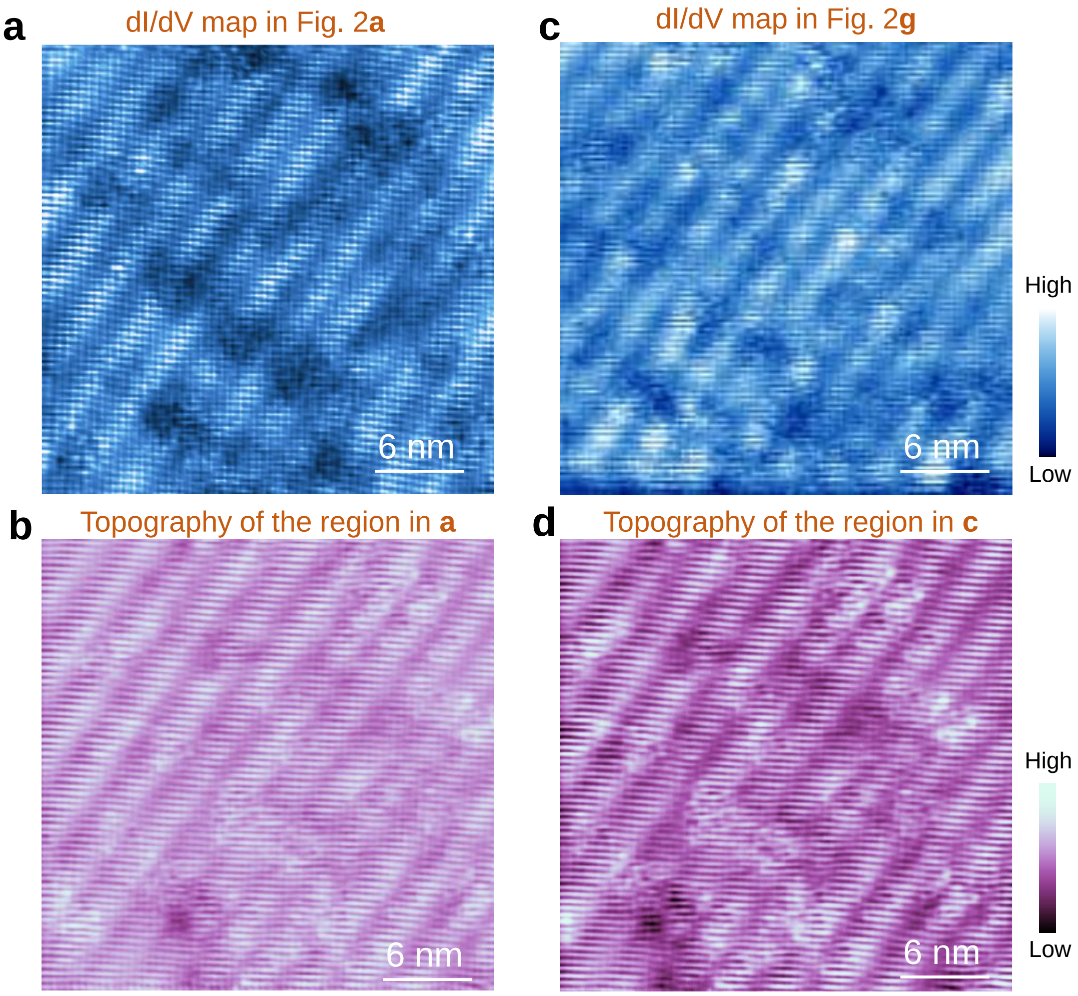

Supplementary Fig. 3: Comparison of CDW stripes in dI/dV maps and STM topographies. a,b, dI/dV map (**a**) and corresponding STM topography (**b**) of the region in Fig. 2**a**. **c,d,** dI/dV map (**c**) and corresponding STM topography (**d**) of the region in Fig. 2**g**. While the CDW stripes in dI/dV maps look vague and disordered due to the presence of defects, pronounced and well-ordered CDW stripes can be resolved in the topographies.

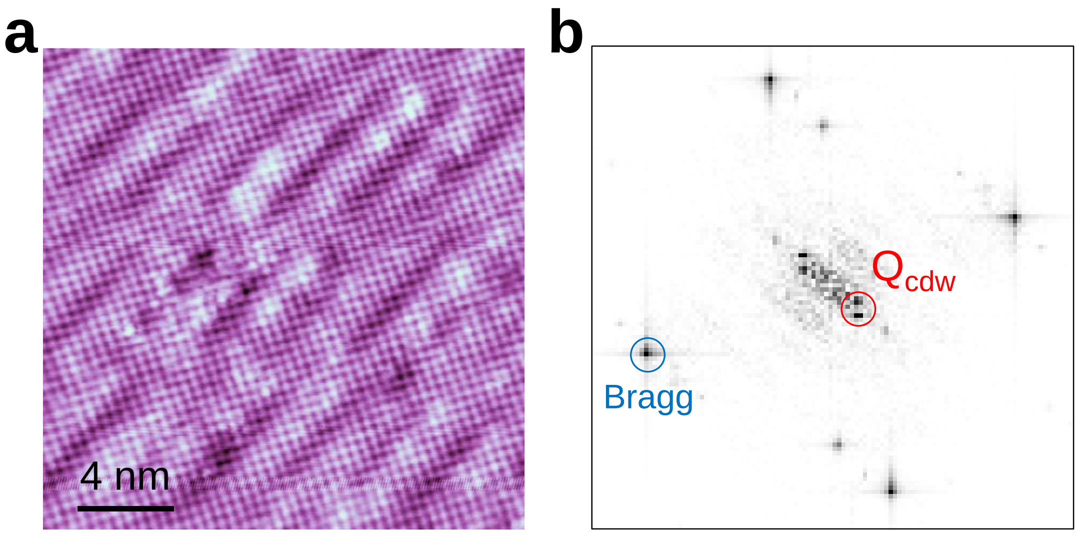

Supplementary Fig. 4: STM image and the corresponding FT image of CoSi (001) surface under 80 K. **a,** 20 nm × 20 nm STM topography. Scanning parameters: V_s=-200 mV, I_t=0.1 nA. **b,** FT image of **a**. The CDW stripes are less ordered, showing that the local defects affect the ordering of the stripes under high temperature.

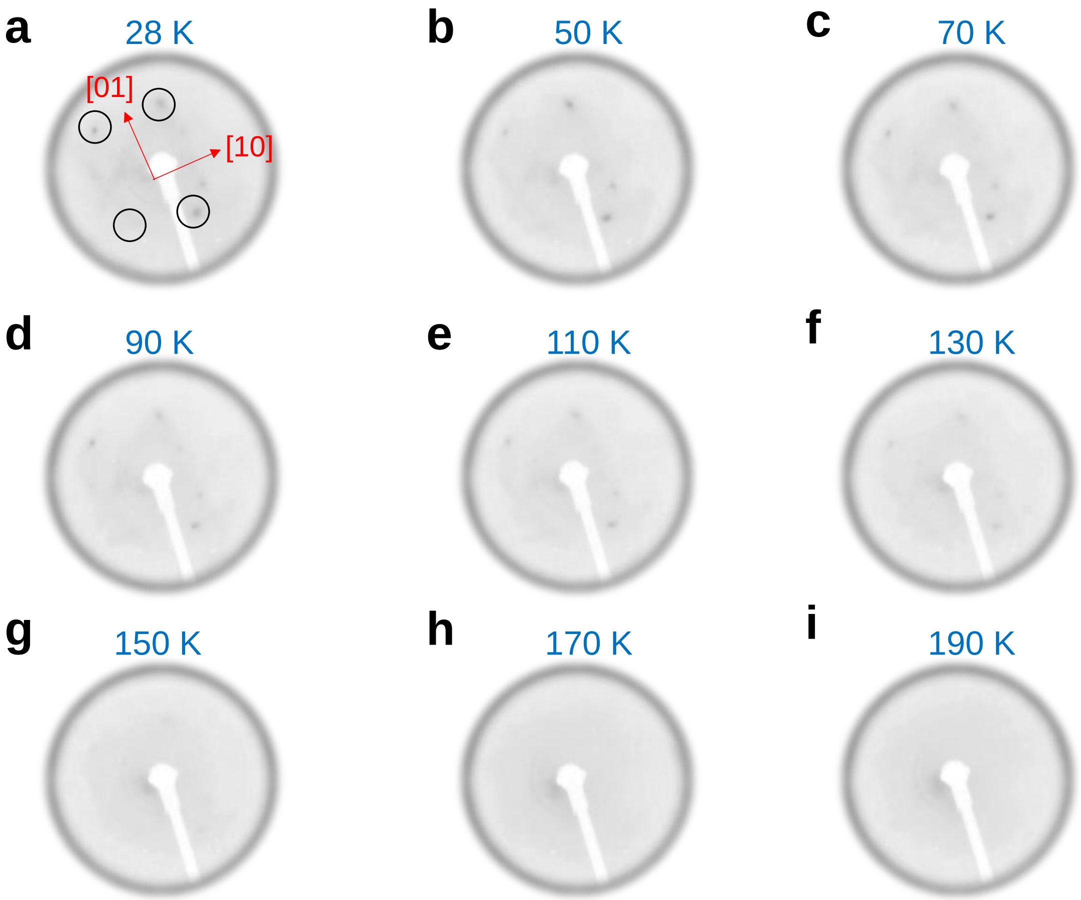

Supplementary Fig. 5: LEED pattern of the CoSi (001) surface under different temperatures. Temperature dependent LEED pattern showing the diffraction spots of the CDW pattern taken under 28 K, 50 K, 70 K, 90 K, 110 K, 130 K, 150 K, 170 K, and 190 K, respectively. Beam energy: 14 eV. The CDW spots disappear above 150 K.

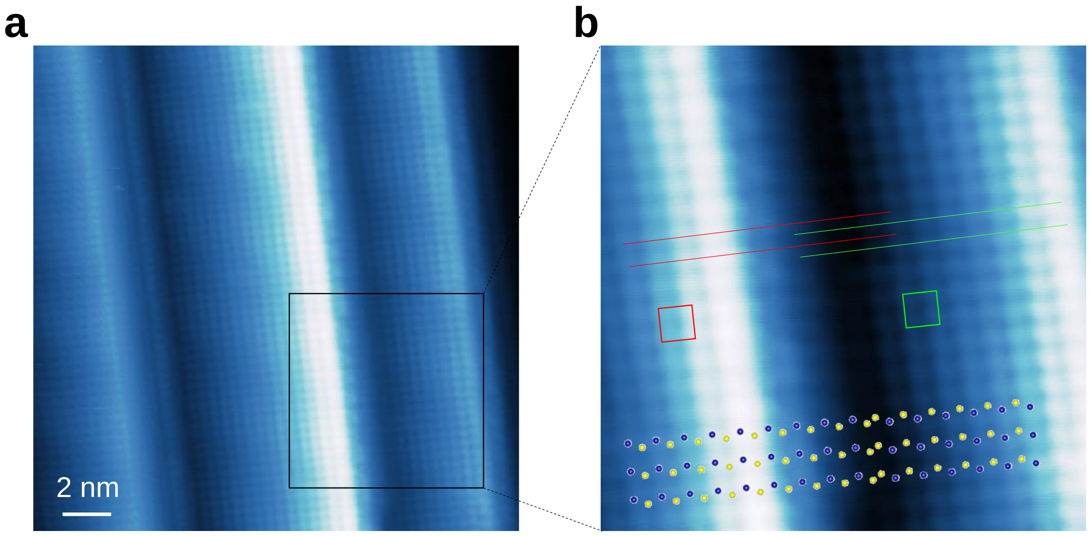

Supplementary Fig. 6: Atomic structure change across odd steps. a, STM image (20 nm × 20 nm) showing the square Co lattice on the terraces. The scanning area is the same as Fig. 3**e**. **b,** Zoom-in image of the black square in **a**. The surface Co and Si atoms are overlaid. The atomic rows on terraces with different index are not aligned with each other as outlined by the red and green lines, indicating the glide-mirror symmetry of these terraces. The unit cells of the upper and lower terraces are outlined by red and green squares, respectively. Scanning settings: V_s=-200 mV, I_t=0.1 nA.

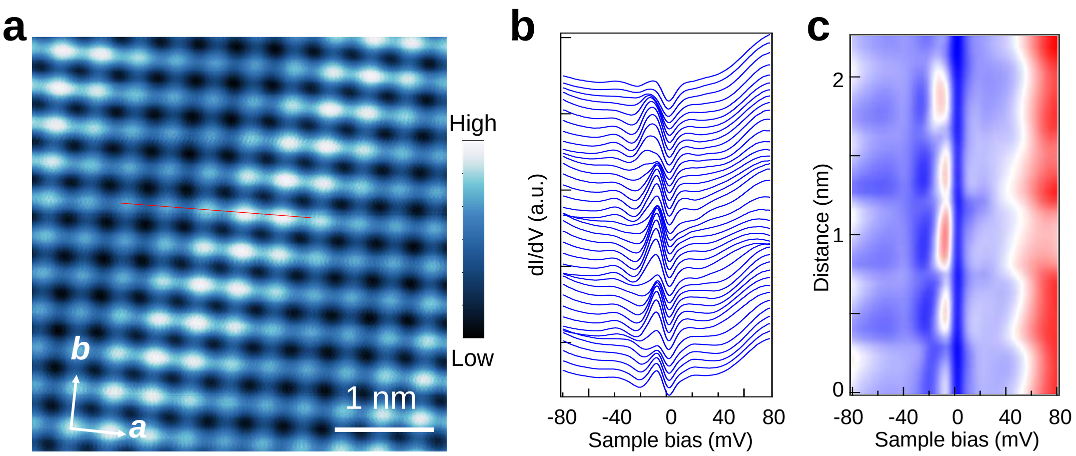

Supplementary Fig. 7: STM image and dI/dV spectra inside the unit cell along the [10] direction of the surface. **a,** Atomically-resolved STM image showing square CoSi lattice and CDW stripes. **b,c,** Waterfall plot and intensity map of the dI/dV curves along the red line in **a**, showing strong periodic modulation of the dI/dV spectra of the lattice.

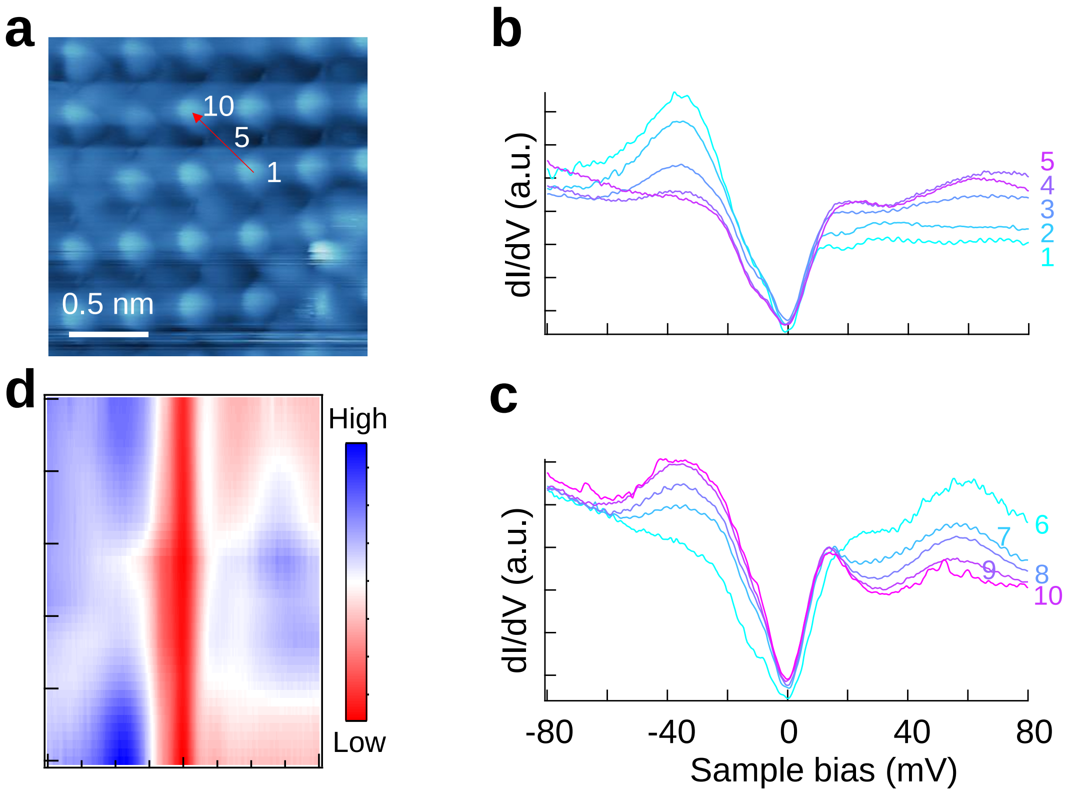

Supplementary Fig. 8: STM image and dI/dV spectra inside the unit cell along the [11] direction of the surface. **a,** Atomic image of the surface. **b-d,** Stacking plot and intensity map of the spectra along the red arrow in **a**, showing strong intra-cell charge modulation.

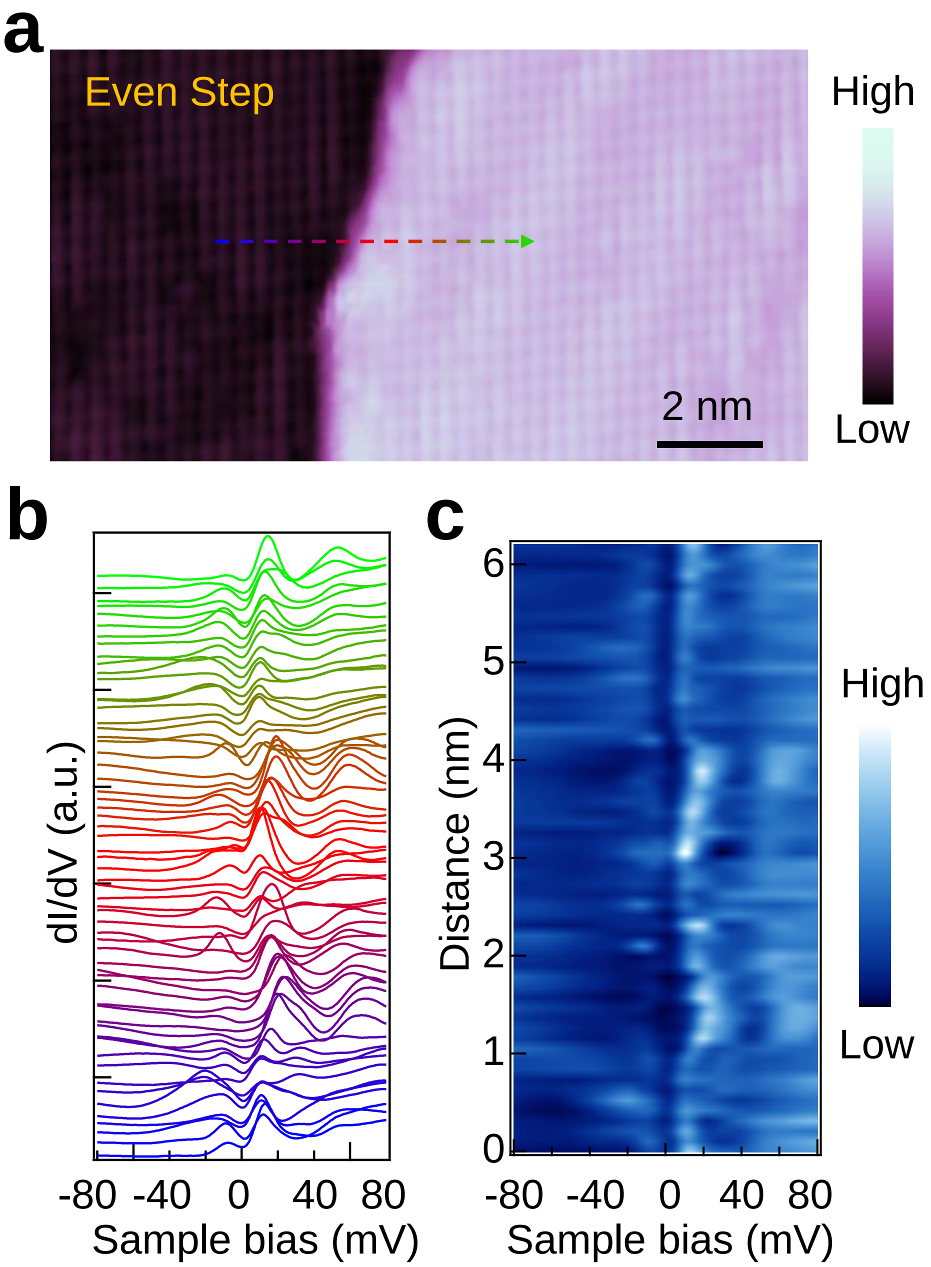

Supplementary Fig. 9: Spatial evolution of dI/dV spectra across an even step. a, STM image of an even step. Scanning settings: V_s=50 mV, I_t=0.3 nA. **b,c,** Waterfall plot and intensity map of dI/dV spectra along the dashed arrow in **a**. On both lower and upper terraces, the asymmetric CDW coherence peaks show larger spectral weight at the positive bias. The asymmetry of the CDW coherence peaks keeps the same characteristics across the even step.

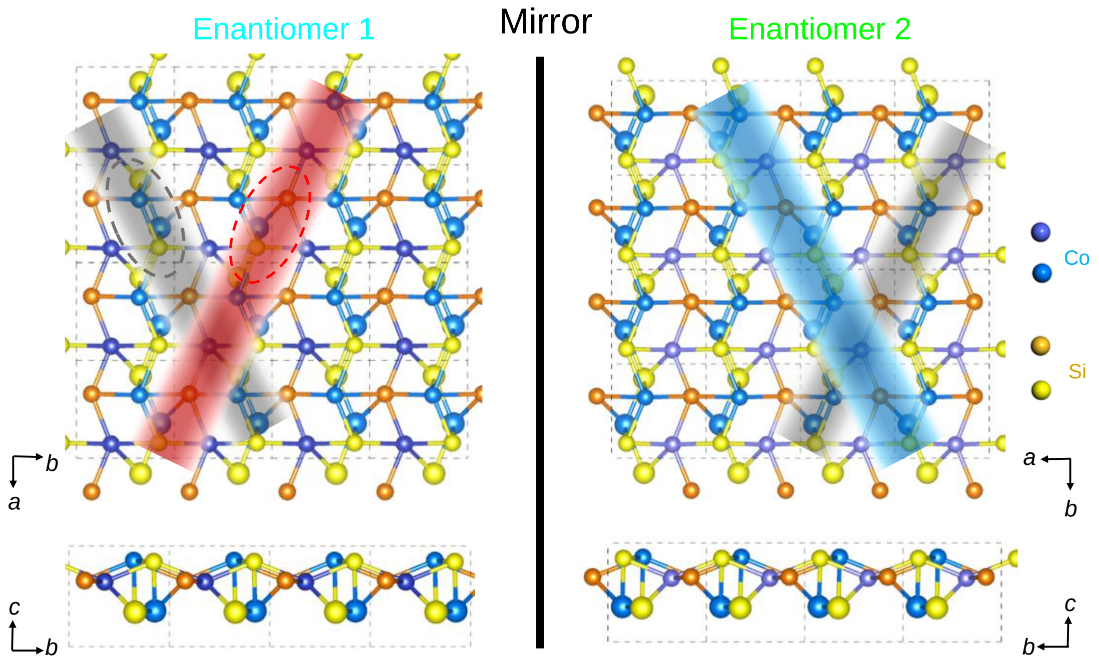

Supplementary Fig. 10: Schematic of electron hopping paths in the two crystal enantiomers of CoSi. Left panel, atomic structures of the top- (upper panel) and side-views (lower panel) of the top three sublayers of enantiomer 1. The red line outlines the electron hopping path of the CDW order, following an atomic sequence of Co_1-Co_2-Si_2-Si_1-Co_1-Co_2…. If we only consider the top two sublayers, an equivalent path is labeled by a grey line. However, they become inequivalent if the third sublayer is considered. In particular, Co_3 lies below the interval between Si_2 and Si_1 along the red path, while it sits below the interval between Si_1 and Co_1 along the grey path, as highlighted by the red and grey dashed ellipses. As a result, the electron hopping path is unidirectional. Right panel, atomic structures of the top- (upper panel) and side-views (lower panel) of the top three sublayers of enantiomer 2. The blue line outlines the electron hopping path of the CDW order.

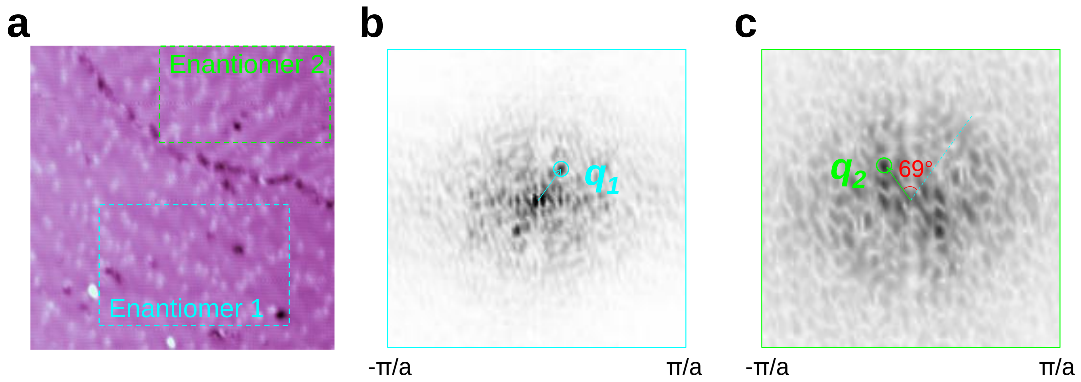

Supplementary Fig. 11: FT images of the different CDW wavevector in different enantiomers. a, STM image of the two enantiomers, which is reproduced from Fig. 4**c**. **b,c,** FT image of enantiomer 1 (**b**) and 2 (**c**) as outlined by the blue and green dashed rectangles in **a**. CDW wave vectors $\boldsymbol{q_1}$ and $\boldsymbol{q_2}$ can be extracted from the FT images for the two enantiomers. The crystal lattice of the enantiomer 1 region shows a 90° rotation clockwise compared with that in Fig. 1. The angle between $\boldsymbol{q_1}$ and $\boldsymbol{q_2}$ is ~69°. The two CDW patterns show mirror symmetry with respect to the horizontal axis.

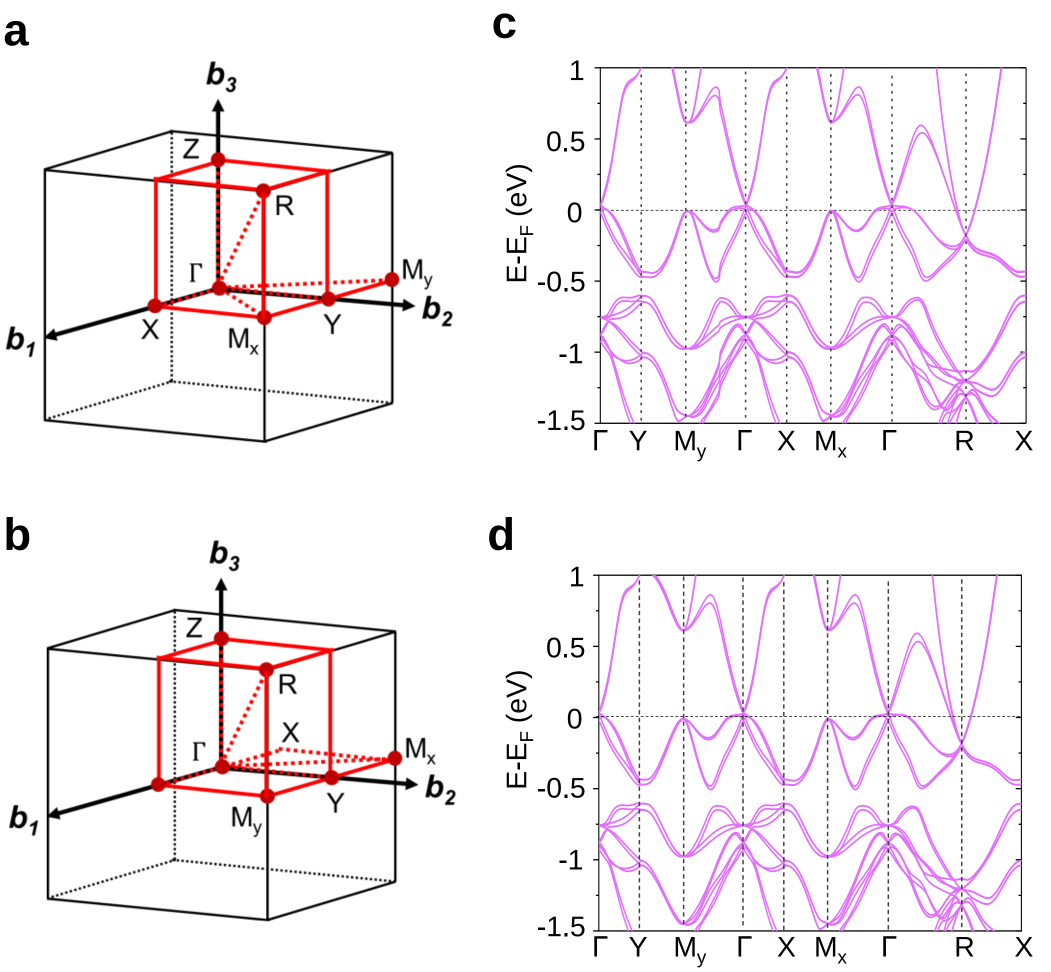

Supplementary Fig. 12: Electronic bandstructures of the two crystal enantiomers. **a,b,** Bulk Brillouin zone of enantiomers 1 (**a**) and 2 (**d**). **c,d,** Theoretical electronic bandstructures, with consideration of spin-orbit coupling, of the CoSi bulk crystal along high-symmetry lines of enantiomers 1 (**c**) and 2 (**d**).

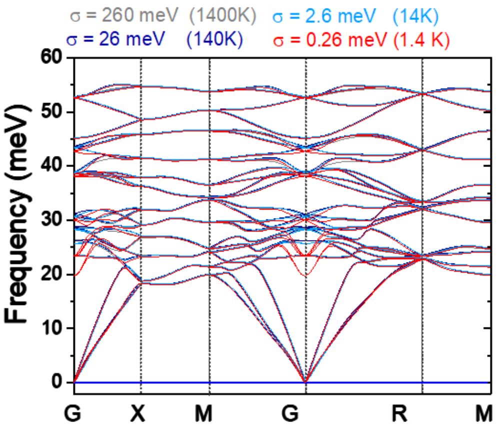

Supplementary Fig. 13: Phonon dispersion of bulk CoSi for several smearing factors using the Fermi-Dirac function as smearing function. Softening of phonon modes are visible around the Gamma points but without emergence of imaginary frequencies, which indicates a relative weak electron-phonon coupling.
